# Too complex, too busy, yet paid the same: why university tertiary hospitals need a new model

**DOI:** 10.3325/cmj.2026.67.22

**Published:** 2026-02

**Authors:** Mislav Puljević

**Affiliations:** University Hospital Centre Zagreb, University of Zagreb School of Medicine, Zagreb, Croatia

## Abstract

University tertiary hospitals in Croatia carry a disproportionate share of complex care, teaching, and research responsibilities, yet they are reimbursed under the same Diagnosis-Related Group framework as smaller secondary hospitals. This structural misalignment contributes to workforce strain, physician migration, and inefficiencies in patient flow, while challenging long-term system sustainability. This narrative review and policy analysis synthesizes biomedical literature, international policy reports, and national documents published between 2000 and 2024 to examine workforce shortages, burnout, migration patterns, financing models, and the Croatian context. Croatia reports 3.4 physicians per 1000 inhabitants compared with the European Union average of 4.1, while maintaining a highly centralized referral structure. Burnout prevalence among physicians is estimated at 30–50%, and in 2021 approximately 7% of Croatian physicians applied for certificates enabling employment abroad. Germany, France, the United Kingdom, Scandinavian countries, and Canada have introduced differentiated financing mechanisms that compensate tertiary hospitals for case complexity, referral flows, and academic responsibilities. A pilot Workload Index model is proposed to align reimbursement with case mix, teaching load, referral inflow, and occupational risk exposure. Linking financing to measurable workload indicators may support fairer resource allocation, workforce protection, and improved access to complex care within Croatia’s tertiary health services.

Health systems globally are undergoing profound transformation, shaped by demographic aging, rising patient expectations, increasing technological complexity, and unprecedented workforce pressures. Among the most affected institutions are university tertiary hospitals, which occupy a unique and irreplaceable position in health care delivery. These institutions not only provide the highest level of specialized clinical services but also serve as hubs for medical education and biomedical research. Their dual role as both clinical and academic centers makes them particularly vulnerable to imbalances in financing and workforce policy.

The Organization for Economic Co-operation and Development (OECD) and World Health Organization (WHO) project persistent shortages of health professionals across member states in the coming decade (1,2). Beyond absolute numbers, the qualitative distribution of workload is a central issue. University tertiary hospitals absorb the most complex referrals, often serving as the last resort for patients across entire nations. In Croatia, this burden is concentrated in a single university tertiary hospital of the highest category, which functions simultaneously as the primary center for advanced cardiovascular, oncological, and surgical care, while also bearing responsibility for undergraduate and postgraduate medical education. Despite this reality, financing is based on uniform diagnosis-related group (DRG) tariffs that do not adjust for complexity, volume, or academic mission (3).

Workforce stress further undermines sustainability. Burnout is one of the most pressing occupational hazards in medicine, with prevalence in European studies ranging between 30% and 50% (4). The WHO formally recognized burnout as an occupational phenomenon in the ICD-11 (5).

Hospital financing is a decisive policy lever. The DRG system, while improving transparency and efficiency, is widely recognized as insufficient for capturing the full cost and complexity of tertiary care (3). To mitigate these shortcomings, European countries have developed supplementary financing mechanisms. By contrast, Croatia continues to apply a uniform DRG reimbursement system across all hospital categories. This creates an unsustainable paradox: Croatia’s university tertiary hospital carries the heaviest clinical and academic load yet receives identical financial treatment as smaller secondary hospitals. This imbalance fuels workforce dissatisfaction, accelerates burnout, and undermines both clinical and academic sustainability.

Furthermore, weaknesses in primary, secondary, and palliative care exacerbate pressure on tertiary hospitals. Insufficient development of community-based and regional care systems results in inappropriate admissions to tertiary centers, where resources are already strained. Patients requiring long-term palliative care often occupy acute tertiary beds, which prevents timely access for patients with urgent and complex conditions. This structural bottleneck illustrates the interconnectedness of all care levels and the necessity of a system-wide reform.

Against this background, the present study seeks to:

1. Analyze the unique role of university tertiary hospitals in Croatia’s health system.

2. Compare international models of tertiary hospital financing and workforce policy.

3. Propose a pilot model that aligns financing, workload monitoring, and workforce protection with actual institutional responsibilities, thereby improving retention, reducing burnout, and strengthening systemic resilience.

## Methods

This study was designed as a narrative review and policy analysis aimed at situating the role of university tertiary hospitals in Croatia within the broader European and international context. A narrative review was chosen because of its capacity to synthesize evidence from heterogeneous sources, ranging from peer-reviewed biomedical studies to international policy reports and national gray literature, which together provide a more comprehensive understanding of financing and workforce challenges.

A structured search strategy was applied to PubMed/MEDLINE for the period January 2000 to June 2024. The search string combined terms such as “tertiary hospital,” “academic medical center,” “university hospital,” “financing,” “funding,” “Diagnosis Related Groups,” “workload,” “case mix,” “burnout,” “migration,” and “policy reform.” Boolean operators and Medical Subject Headings were employed to increase sensitivity. Only publications in English were included. To complement the search results, the OECD iLibrary, the WHO Global Health Observatory, Eurofound, and the European Observatory on Health Systems and Policies were searched for policy reports and data. Gray literature, including Croatian health ministry documents, professional society position papers, and EU workforce mobility reports, was also considered.

Eligibility was determined by relevance to hospital financing, workload distribution, workforce shortages, burnout, or physician migration. Non-English publications, opinion pieces without empirical basis, and studies not related to hospital workforce or financing issues were excluded.

Two reviewers independently screened abstracts and full texts, and extracted data into thematic matrices. The key domains defined *a priori* were 1) workforce shortages and projections, 2) burnout and occupational risks, 3) migration and retention, 4) financing models for tertiary hospitals, and 5) the Croatian context. A thematic synthesis approach was used to integrate findings, while case studies from Germany, France, the United Kingdom, Scandinavia, Canada, and Romania were examined in depth to provide comparative perspectives. Triangulation of data from peer-reviewed studies, international policy reports, and national gray literature was employed to ensure validity and consistency of findings.

### Comparative case study approach and contextual controls

To minimize naïve transfer across dissimilar systems, we used a structured, focused comparison. For each country case (Germany, France, the United Kingdom, Scandinavia, Canada, Romania), we extracted a common dimension set: 1) hospital payment regime (DRG base and add-ons); 2) legal status and earmarked funding of university/teaching hospitals; 3) referral legislation and cross-regional patient flows; 4) governance centralization and purchaser-provider split; 5) path-dependency markers (timing/sequence of reforms); and 6) political tradition/coalition patterns affecting health budgets. Findings are presented on a common template to make differences visible. Policy inferences for Croatia are restricted to mechanism-level analogies (eg, earmarked teaching top-ups; specialized commissioning) and explicitly conditioned on Croatia’s centralized referral structure and single top-tier university hospital configuration.

### Stakeholder framings and policy narratives in Croatia

We complemented the substantive domains (financing, workforce, burnout) with a narrative framing lens. Drawing on policy documents, professional society statements, hospital reports, and media commentaries, we conducted a structured “who-says-what” scan to identify dominant narratives and marginalized voices. We coded references to tertiary hospitals as (a) cost burden (efficiency/DRG focus), (b) public good (education, rare/complex care, positive externalities), (c) regional equity (referral flows), and (d) workforce protection (occupational risks/teaching load). We then mapped framings to stakeholder groups (Ministry/payers, university hospitals, regional hospitals, professional chambers, and patient groups) and noted which narratives dominate public debate. This clarified how problem definitions shape feasible solutions and why uniform DRG tariffs persist despite the complexity borne by university tertiary hospitals.

## Results

### Workforce shortages

The OECD and WHO project persistent shortages of health professionals across member states in the coming decade (1,2). Croatia exemplifies this trend, with only 3.4 physicians per 1000 inhabitants, a figure significantly below the European average (14). Germany and Scandinavian countries maintain higher densities (4.3-4.5 per 1000), while France is slightly below average at 3.2, and Poland reports one of the lowest levels in the EU with only 2.4 physicians per 1000 (2,14). These discrepancies reveal structural imbalances in workforce distribution that extend beyond absolute numbers.

Croatia falls into the group of below-average countries, despite relying on a highly centralized tertiary care system. This mismatch between demand and capacity threatens the sustainability of its national referral pathways (Table 1).

**Table 1:** The number of physicians per 1000 inhabitants (selected countries, 2022)

Country	Physicians/1000	Comparison to the EU average (4.1)
Germany	4.5	Above average
Denmark	4.4	Above average
Sweden	4.3	Above average
Croatia	3.4	Below average
France	3.2	Below average
Poland	2.4	Significantly below average

### Burnout and occupational risks

Shortages are compounded by burnout, which has become a critical occupational hazard in health care. Studies consistently report the prevalence of burnout among physicians between 30% and 50%, with the highest rates among intensivists and anesthesiologists (4). Burnout is characterized by emotional exhaustion, depersonalization, and a reduced sense of professional accomplishment. In recognition of its widespread impact, the WHO included burnout in the ICD-11 as an occupational phenomenon rather than a medical diagnosis (5). Eurofound surveys confirm that health care is among the three sectors with the highest rates of work-related stress and absenteeism in Europe (6).

In Croatia, the problem is exacerbated by discrepancies between real occupational risk and formal recognition. For instance, physicians performing procedures under x-ray guidance – such as pacemaker implantation and catheter ablation – are often not classified administratively as operating theater staff. As a result, they are excluded from certain protections and benefits despite tangible exposure to ionizing radiation (7). This disconnect between actual workload and bureaucratic classification illustrates systemic flaws in how occupational risks are regulated.

### Migration and retention

In 2017, Romania introduced major salary increases for health professionals, reaching 70%-200% depending on rank, which temporarily slowed outward migration (8). Nevertheless, structural issues persisted, such as uneven regional distribution of staff and high workloads in tertiary centers. Similar dynamics have been reported in Poland, where approximately 10% of all licensed physicians practice abroad, most commonly in Germany and the United Kingdom (16). In Croatia, the Medical Chamber reported that around 1000 physicians – equivalent to roughly 7% of the total workforce – had applied for certificates of good standing to enable employment abroad in 2021 alone. While wages are a major factor, qualitative aspects such as working conditions, workload intensity, and structured career pathways remain strong motivators for emigration.

### Financing models for tertiary hospitals

Hospital financing plays a decisive role in shaping institutional sustainability. The DRG model is the dominant reimbursement framework across Europe, but it is widely criticized as inadequate for capturing the true costs of highly complex care delivered in tertiary hospitals (3).

Several countries have addressed these shortcomings through differentiated financing. In Germany, supplementary DRG payments (*Zusatzentgelte*) are applied to high-cost interventions and advanced technologies (9). France allocates earmarked budgets to its university hospitals to sustain their dual missions of teaching and research (10). The United Kingdom employs specialized commissioning, a national system designed to guarantee funding for rare and complex conditions, thereby protecting local budgets from unsustainable burdens (11). In Scandinavia, regional referral pathways are embedded into legislation, ensuring financial transfers when tertiary hospitals provide services to patients from outside their administrative region (12). In Canada, teaching supplements are integrated into the budgets of Academic Health Science Centres, directly acknowledging their academic responsibilities (13). These adaptations (Table 2) demonstrate that Croatia’s uniform DRG system is increasingly an outlier in Europe.

**Table 2:** Selected financing adaptations for tertiary hospitals

Country	Mechanism	Purpose
Germany	*Zusatzentgelte* (supplementary Diagnosis-Related Groups)	Compensate high-cost interventions
France	Earmarked *Centre Hospitalier Universitaire* budgets	Sustain teaching and research
UK	Specialized commissioning	Fund rare and complex conditions
Scandinavia	Regionalized referral financing	Support cross-regional tertiary care
Canada	Teaching supplements	Recognize academic responsibilities

### Croatian context

In contrast to these differentiated models, Croatia continues to apply uniform DRG reimbursement across all hospital categories. Its only university tertiary hospital of the highest category functions simultaneously as the national referral center for highly complex interventions and as the core academic institution responsible for undergraduate and postgraduate training. Yet, it receives identical financial treatment as smaller secondary hospitals (3,15).

This creates an unsustainable paradox: the most burdened institution, both clinically and academically, receives no structural recognition of its additional responsibilities. Combined with shortages of staff, high rates of burnout, and persistent migration, this uniformity undermines the sustainability of tertiary care.

The situation is aggravated by weaknesses in primary, secondary, and palliative care. Many patients requiring chronic or end-of-life management occupy tertiary beds, which prevent timely admission of patients with acute and complex conditions. This inefficiency reflects systemic gaps and highlights the interdependence of all levels of care.

## Discussion

The present analysis demonstrates that Croatia’s university tertiary hospitals operate under systemic constraints that undermine their ability to sustain both clinical and academic missions. While most European countries have gradually recognized the need for differentiated financing models that reflect the complexity of tertiary care, Croatia continues to reimburse its most burdened institutions on the same terms as smaller regional hospitals. This uniformity disregards referral flows, case complexity, teaching responsibilities, and occupational risks, creating inefficiencies that manifest in workforce dissatisfaction, migration, and patient-care bottlenecks.

### Lessons from abroad

In 2017, Romania introduced major salary increases for health professionals, reported to reach 70–200% depending on rank, which temporarily slowed outward migration (8). This lesson is directly applicable to Croatia. While salary increases could provide temporary relief, they would not resolve the core imbalance between institutional responsibilities and reimbursement mechanisms. Without structural realignment, wage reforms risk producing only short-lived improvements while leaving systemic dysfunction intact.

### Toward a new model

Workload Index (WI): provisional specification and funding linkage

The evidence underscores the need for a model that explicitly links financing, workload, and workforce protection. Croatia’s current DRG-based uniformity is increasingly untenable in a system where one institution carries disproportionate clinical and academic responsibilities. The challenge is to design a reform that is both fair and operationally feasible, providing measurable improvements in workforce satisfaction, patient flow, and institutional sustainability (Table 3, Table 4).

**Table 3:** The computation of the Workload Index (WI) from normalized components and conversion into a capped linear funding top-up (illustrative values only)

WI components – composite WI and funding top-up	Weight	Metric (example)	Normalized (0–1)	Contribution (weight × norm.)
Case-mix (average DRG weight)	0.35	1.85	0.80	0.28
Referral inflow (share of out-of-region patients)	0.25	46%	0.46	0.12
Teaching load (students + residents per FTE)	0.25	2.2	0.55	0.14
Fluoroscopy-exposed procedures per MD	0.15	120	0.60	0.09
Composite WI (sum)				**0.63**
Funding top-up (linear; cap 12%)				**0.63 × 12% = 7.6%**

*Abbreviations: DRG – diagnosis-related group; FTE – full-time equivalent; MD – medical doctor.

For transparency and replicability, we present a provisional specification:

WI = 0.35 · (case-mix complexity; mean DRG weight, normalized) + 0.25 · (referral inflow share) + 0.25 · (teaching load per full-time equivalent) + 0.15 · (fluoroscopy-exposed procedures per physician).

Components are z-standardized to [0–1]. Top-ups are linearly linked to WI within a capped budget envelope; weights are calibration targets in the 12-month pilot.

### Expanded pilot model

We propose a pilot model structured around three interconnected pillars: workload monitoring, differentiated incentives, and workforce protection. The cornerstone of this model is the Workload Index, a composite metric designed to capture the multidimensional burden of tertiary hospitals.

The index integrates four domains. Case mix complexity would be quantified through average DRG weights, reflecting the intensity of clinical work. Referral inflow would measure the proportion of patients admitted from outside the local catchment area, an especially critical variable in Croatia’s centralized system. Teaching load would be calculated as the number of undergraduate students, residents, and specialty trainees per full-time faculty member, acknowledging the academic mission of university hospitals. Finally, occupational risk exposure would be quantified by the number of procedures performed annually under fluoroscopy or other ionizing radiation per physician, adjusted for protective measures. Each domain would be normalized and equally weighted, yielding a standardized index ranging from 0 to 1.

Linking reimbursement to this workload index would allow tertiary hospitals to receive financing proportionate to their real burden. In addition to institutional funding, salary supplements would be directed toward staff performing radiation-exposed or high-complexity procedures, while teaching responsibilities would be formally remunerated to reduce reliance on uncompensated academic labor.

Outcome measures are central to the evaluation of the pilot. Staff retention rates would be tracked at 12 and 24 months. Burnout prevalence would be measured annually using validated tools such as the Maslach Burnout Inventory. Patient flow efficiency would be assessed through average length of stay, refusal of admissions due to capacity constraints, and turnover of acute beds. Educational performance, measured by the successful completion of rotations by students and residents, would ensure that academic functions are not neglected.

Governance and oversight must be robust. The Ministry of Health would regulate the pilot and allocate financing, while participating tertiary hospitals would implement the model and report data quarterly. An independent supervisory body, such as the Agency for Quality and Accreditation in Health Care, would audit results and ensure transparency.

The pilot would begin in a single university tertiary hospital, providing an opportunity to refine indicators and resolve implementation challenges. If successful, the model could be expanded to additional tertiary centers and, in a modified form, to regional hospitals handling high referral volumes.

Potential obstacles include increased administrative workload, risk of data manipulation, and financial sustainability. These challenges can be mitigated through digital integration of the workload index into electronic health records, independent audits, and phased budget allocation with predefined caps (Table 5).

**Table 5:** Expanded pilot model for university tertiary hospitals

Component	Key elements	Expected outcomes
Workload monitoring	Case mix, referral inflow, teaching load, occupational risks	Transparent recognition of burden
Differentiated incentives	Funding tied to index; salary supplements for high-risk procedures; remuneration for teaching	Fairer pay, improved retention
Workforce protection	Legal recognition of exposure; benefits linked to actual work; investment in secondary/palliative care	Improved safety, reduced burnout, optimized capacity
Evaluation	Staff retention, burnout prevalence, patient flow, educational outputs	Evidence-based policy refinement
Governance	Ministry of Health regulation; independent supervisory agency	Transparency and accountability
Scalability	Pilot in one tertiary hospital; phased expansion to others	System-wide sustainability

By embedding structural recognition of workload, teaching, and occupational risk into financing, the proposed pilot model addresses the root causes of workforce dissatisfaction and systemic inefficiency (Table 5). Unlike temporary salary increases, this framework offers a durable mechanism for aligning institutional responsibilities with resources. If successfully implemented, it would not only stabilize Croatia’s university tertiary hospitals but could also provide a replicable blueprint for health systems facing similar imbalances between tertiary and secondary care.

### Limitations

Several limitations of this study should be acknowledged. First, as a narrative review and policy analysis, it relies on secondary data sources rather than primary empirical measurements. The accuracy of conclusions depends on the validity and comprehensiveness of published studies, international reports, and national policy documents. Second, while international models offer valuable lessons, their direct transferability to Croatia may be limited by differences in health financing, political will, and administrative capacity. Third, the proposed pilot model, though operationalized with measurable indicators, remains hypothetical until tested in practice. Its feasibility and impact can only be confirmed through implementation and empirical evaluation.

### Future directions

Future research should focus on the empirical testing of the pilot model in a real-world setting. This would involve developing digital tools to automate workload monitoring, conducting prospective studies on burnout and staff retention, and performing cost-effectiveness analyses of differentiated financing. Comparative research between Croatian tertiary hospitals and those in neighboring countries could further contextualize findings and refine the model. Additionally, the role of primary, secondary, and palliative care in alleviating tertiary overload warrants deeper investigation, as strengthening these levels of care is essential for the long-term sustainability of the system.

Ultimately, a reform of hospital financing must be viewed not as an isolated measure but as part of a broader health system transformation. Aligning resources with responsibilities is a necessary step toward ensuring that tertiary hospitals can continue to fulfill their dual mission of providing advanced care and training the next generation of health care professionals.

### Conclusion

This study highlights the structural vulnerabilities of Croatia’s hospital financing model, which applies uniform DRG reimbursement across all hospital categories but disregards the distinct responsibilities of university tertiary hospitals. Comparative international analysis demonstrates that differentiated financing for tertiary centers is a standard feature of advanced health systems, ensuring sustainability for institutions that simultaneously carry referral, teaching, and research obligations.

The Croatian system, in contrast, leaves its sole highest-category tertiary hospital overburdened and under-recognized, a situation that exacerbates workforce shortages, burnout, and migration. By integrating lessons from other countries and tailoring them to Croatia’s specific challenges, the proposed pilot model provides a structured pathway toward reform. Its core innovation – the Workload Index – links financing directly to case complexity, referral inflow, teaching load, and occupational risk exposure. Coupled with differentiated incentives and workforce protection, the model offers a comprehensive framework for aligning resources with responsibilities. If implemented and validated, this pilot has the potential not only to stabilize Croatia’s tertiary hospitals but also to serve as a model for other health systems with similar structural imbalances.

**Table 4:** Primary key performance indicators (KPIs) and 12-month targets for pilot evaluation; baselines are entered from hospital administrative data at pilot initiation

Pilot evaluation KPIs (12-mo targets; baseline to be populated at pilot start)	Baseline	Target (12 mos)	Data source
Physician retention (tertiary hospital)	TBD	**≥93%**	HR/Payroll
Burnout (MBI: high-risk)	TBD	**≤30%**	Validated survey (MBI)
ALOS — complex cases (days)	TBD	**≤8.5**	HIS/BI reports
Denied admissions due to capacity/mo	TBD	**≤50**	Intake logs
Out-of-region patient share	TBD	**Maintain transparency/stability**	HIS/Referral logs
Completed student & resident rotations	TBD	**≥99%**	Academic records

*Abbreviations: MD – medical doctor, MBI – Maslach Burnout Inventory, HR – human resources, TBD – to be decided by provider, ALOS – average length of stay; HIS/BI – hospital information system/business intelligence.
